# Visceral fat-to-muscle ratio guides spontaneous closure in post-sepsis duodenal fistula: a body composition divergence beyond visceral-subcutaneous fat ratio

**DOI:** 10.3389/fnut.2025.1604230

**Published:** 2025-05-30

**Authors:** Qian Tang, Wuhan Li, Xin Xu, Risheng Zhao, Yunzhao Zhao, Zheng Yao

**Affiliations:** ^1^Department of General Surgery, Jiangning Hospital, Nanjing, Jiangsu, China; ^2^Department of General Surgery, Anhui Provincial Hospital, Hefei, Anhui, China

**Keywords:** duodenal fistula, body composition, sepsis, visceral fat area, spontaneous closure

## Abstract

**Background:**

This study aimed to investigate whether body composition parameters (visceral fat area/total abdominal muscle area index, VFA/TAMAI; visceral-to-subcutaneous fat ratio, VFA/SFA) predict spontaneous closure in duodenal fistula patients after sepsis resolution.

**Methods:**

A multicenter retrospective study enrolled 104 duodenal fistula patients with controlled sepsis treated between 2019 and 2025. Standardized management included infection control and nutritional support. Restricted cubic spline regression identified optimal cutoffs for VFA/TAMAI and VFA/SFA. Cox proportional hazards models evaluated associations with 90-day spontaneous closure, with inverse probability treatment weighting (IPTW) and propensity score matching (PSM) addressing confounding.

**Results:**

52.9% (55/104) achieved spontaneous closure within 90 days (median time: 31 days). Elevated VFA/TAMAI (≥3.20, 31.7% patients) independently predicted reduced closure likelihood (adjusted HR = 0.59, 95%CI:0.42–0.85, *P* = 0.004), while VFA/SFA showed no prognostic value (*P* > 0.05). Incorporation of VFA/TAMAI significantly improved predictive accuracy (C-index increased from 0.67 to 0.72, *P* = 0.013). Sensitivity analyses confirmed VFA/TAMAI≥3.20 remained predictive after IPTW adjustment (HR = 0.38, 95%CI:0.17–0.84) and PSM (HR = 0.39, 95%CI:0.19–0.83).

**Conclusion:**

VFA/TAMAI serves as a robust predictor of spontaneous closure in post-sepsis duodenal fistula, with the threshold of 3.20 identifying high-risk patients requiring intensified nutritional-metabolic interventions. These findings highlight body composition monitoring as a critical adjunct to conventional nutritional management.

## 1 Introduction

Nutrition is a critical determinant in the healing of duodenal fistulas, profoundly impacting patient recovery and outcomes (1–3). Adequate nutritional support provides the essential substrates for tissue repair, maintains immune competence, and bolsters overall patient resilience during the prolonged treatment of fistulas (4). However, in patients recovering from sepsis, even when adequate nutrition is guaranteed, persistent metabolic disturbances can occur (5). Sepsis induces a hypermetabolic and hypercatabolic state characterized by increased energy expenditure, protein breakdown, and insulin resistance (6). These metabolic derangements may persist beyond the acute phase of sepsis, leading to significant alterations in body composition despite adequate nutritional provision (7). Patients may experience muscle wasting and changes in fat distribution, reflecting underlying inflammatory processes and metabolic stress (8).

The interplay between inflammation and body composition is crucial in understanding the healing outcomes of gastrointestinal fistulas (9). Persistent inflammation and metabolic abnormalities can compromise wound healing mechanisms, leading to prolonged recovery times or failure of fistula closure, even when nutritional support appears adequate (10). Therefore, assessing body composition changes and their association with inflammatory status can provide valuable insights into patient prognosis.

Given these insights, we hypothesize that body composition parameters could serve as predictors of spontaneous closure in patients with duodenal fistulas who have recovered from sepsis. To investigate their influence on fistula closure, this study included two common indicators reflecting body composition: the visceral fat area (VFA) to total abdominal muscle area index (TAMAI) ratio, and the VFA to subcutaneous fat area (SFA) ratio (11, 12). By exploring these relationships, we seek to enhance our understanding of the factors influencing healing outcomes in this challenging patient population and potentially identify new prognostic indicators to guide clinical management.

## 2 Materials and methods

### 2.1 Patients

This retrospective study was conducted at two centers, in accordance with the principles outlined in the Declaration of Helsinki. The study focused on patients who met the following criteria: (1) a duodenal fistula, and (2) a history of sepsis at admission, followed by successful septic control. Patients treated at the participating centers between January 2019 and January 2025 were included in the analysis. Prior to 2022, glutamine was widely used to support intestinal mucosal healing at Center A. In our analysis (not published yet), glutamine was found to play a role in mucosal healing. Consequently, Center A included patients from 2022 to 2025, while Center B included patients from the entire study period, spanning 2019 to 2025. The exclusion criteria included patients under 18 years of age, those with incomplete data, patients with entero-atmospheric fistulas, and those with fistulas located outside the duodenum. Patients were followed until definitive surgery, with a minimum follow-up of 3 months after infection control. The primary outcome measured was spontaneous closure during preoperative treatment.

### 2.2 Treatment of fistula

The clinical management of duodenal fistulas is structured around six fundamental elements: infection control, nutritional support, wound management, fistula evaluation, determination of surgical timing, and therapeutic strategies (13). Sepsis management constitutes the cornerstone of the treatment protocol. For management of infectious foci, percutaneous abdominal drainage served as the primary intervention, while emergency laparotomy was reserved for cases with imaging-confirmed non-drainable abscesses refractory to antibiotic therapy. Notably, approximately 20% of cases presented with intra-abdominal hemorrhage, for which transcatheter arterial embolisation was prioritized, with surgical intervention implemented only after embolisation failure.

Nutritional protocols prescribed a daily caloric intake of 30 kcal/kg, employing a phased approach: total parenteral nutrition (PN) commenced immediately post-admission, transitioning to full enteral nutrition (EN) via nasojejunal tube after infection control. EN infusion was initiated at 20 mL/h, with daily increments of 10 mL/h. Autologous digestive fluid reinfusion was implemented for high-output fistulas (>500 mL/day). Treatment protocols mandated maintenance of hemoglobin above 70 g/L and albumin above 30 g/L, with weekly transfusions categorized as intermittent component therapy when required for target maintenance. Definitive surgery required fulfillment of multiple criteria: ≥1 month of clinical stability, BMI ≥18 kg/m^2^, adequate performance status, hemoglobin ≥100 g/L, albumin ≥30 g/L, and ≥3 months post-source control (11, 14).

### 2.3 Spontaneous closure

During the preoperative treatment period of at least 3 months, gastroenterography was performed upon cessation of intestinal fluid drainage. If gastroenterography did not visualize the fistula, iodixanol was injected via the drainage tube under X-ray guidance. Non-visualization of the duodenum indicated spontaneous closure. Patients were discharged, progressively resuming oral intake.

### 2.4 Data collection

Upon admission, enhanced computed tomography (CT) and gastrointestinal X-ray series were conducted to identify the location and characteristics of the fistula. Gastroscopy was performed during naso-intestinal tube placement, during which the endoscopic fistula diameter was measured, with 2 cm serving as the threshold (15). CT scans were conducted at intervals no longer than seven days during sepsis. Laboratory tests were routinely performed every 2 days for patients with sepsis and every 4 days once sepsis was controlled. The VFA, TAMAI, and SFA values were derived from the most recent CT scan of the patient before sepsis resolution. VFA/SFA and VFA/TAMAI ratios were evaluated using abdominal CT images processed with Image J software (NIH, Bethesda, MD, USA). For each patient, two consecutive axial CT images at the level of the inferior endplate of the L3 lumbar vertebra were processed and averaged. The Sequential Organ Failure Assessment (SOFA) score was evaluated daily. Bilirubin levels above 33 μmol/L (16), an oxygen index ranging from 200 to 300 (16), and creatinine levels exceeding 171 μmol/L (16) indicated hepatic, respiratory, and renal dysfunction, respectively. Sepsis and septic shock were defined according to the Third International Consensus Definitions (17). The absence of infection signs, such as fever and chills, along with the normalization of procalcitonin levels, was considered indicative of infection control. Sepsis, widely accepted as organ dysfunction caused by infection, is considered controlled when infection control is achieved, regardless of any persistent organ dysfunction. Data collected included demographic information, laboratory test results, fistula characteristics, condition on admission, condition at infection control, and comorbidities.

### 2.5 Statistical analysis

Statistical analyses were performed using SPSS software (version 26.0, IBM Corp.) and R (version 4.4.2, R Project for Statistical Computing). To determine the optimal cutoffs of VFA/TAMAI and VFA/SFA associated with spontaneous closure, we utilized restricted cubic spline regression analyses with three knots placed at the 10th, 50th, and 90th centiles respectively. The cutoff points were identified where significant changes in the slope of the spline functions occurred, indicating a change in the association with spontaneous closure. The effect of VFA/TAMAI and VFA/SFA on spontaneous closure was evaluated using Cox proportional hazards regression models. Hazard ratios (HRs) and 95% confidence intervals (CIs) were calculated to assess the strength and direction of the associations. Continuous variables were analyzed using the Mann-Whitney U test, whereas categorical variables were assessed using Fisher's exact test. Kaplan-Meier survival analyses were conducted to estimate the cumulative incidence of spontaneous closure over time, stratified by the identified cutoff values of VFA/TAMAI and VFA/SFA. Sensitivity analyses were performed using inverse probability of treatment weighting (IPTW) to address any imbalance in baseline characteristics between groups defined by body composition indicators with statistical significance in the above analysis. This method creates a weighted pseudo-population in which the distribution of measured baseline covariates is independent of the exposure, thereby reducing bias in estimated treatment effects. Variables with a standardized mean difference (SMD) >0.2 were adjusted for in the IPTW models to ensure adequate balance. A 1:1 propensity score matching (PSM) with a tolerance level of 0.1 was used to validate the results of Sensitivity analyses. A *p* < 0.05 was considered statistically significant for all analyses.

## 3 Results

### 3.1 Clinical characteristics

A total of 166 patients were initially enrolled in this study. Exclusions were made for patients under 18 years of age (*n* = 1), those with incomplete data (due to being transferred to another hospital and loss to follow-up, *n* = 14), patients with enteroatmospheric fistulas (*n* = 23), and patients with fistula outside of duodenum (*n* = 24). The remaining 104 participants were included in the analysis. All enrolled patients ultimately recovered and were discharged, with no reported recurrence of fistulas after discharge. The median age of the cohort was 48 years (interquartile range [IQR]: 36–63 years), and the median BMI at the time of septic control was 20.1 kg/m^2^ (IQR: 18.5–21.9 kg/m^2^). The average time from fistula occurrence to infection control was 39 days (IQR: 32–45 days). The fistulas were caused by tumors (*n* = 33), ulcers (*n* = 28), trauma (*n* = 36), and other factors (*n* = 7, including 5 due to pancreatitis and 2 due to biliary surgery). The fistulas were primarily located at the stump (*n* = 41), bulb (*n* = 33), descending portion (*n* = 25), and horizontal portion (*n* = 5) (Table 1).

**Table 1:** Characteristics of the patients.

**Characteristics**	**Overall population**
**Demographic data**
Male, No. (%)	64 (61.5)
Age, years; (median, IQR)	48 (36–63)
BMI, kg/m^2^, (median, IQR)	20.1 (18.5–21.9)
**Fistula characteristics**
**Location, No. (%)**
Stump	41 (39.4)
Bulb	33 (31.7)
Descending part	25 (24)
Horizontal part	5 (4.8)
**Etiology, No. (%)**
Tumor	33 (31.7)
Ulcer	28 (26.9)
Trauma	36 (34.6)
Others	7 (6.7)
**Output**
< 500 mL/day	7 (6.7)
≥ 500 mL/day, and < 1,000 mL/day	35 (33.7)
≥ 1,000 mL/day	62 (59.6)
Interval from fistula occurred to admission, days (median, IQR)	19 (12-22)
Interval from fistula occurred to infection control, days (median, IQR)	39 (32-45)
Infection invading the retroperitoneum, No. (%)	55 (52.9)
Requirement for emergency laparotomy, No. (%)	32 (30.8)
Abdominal bleeding occurred during the treatment process, No. (%)	34 (32.7)
Endoscopic fistula diameter >2 cm, No. (%)	17 (16.3)
**Condition on admission**
Hemoglobin, g/L; (median, IQR)	81 (64–91)
Albumin, g/L; (median, IQR)	29 (26–32)
Platelet, 10^9^/L; (median, IQR)	103 (76–147)
Procalcitonin, ng/mL; (median, IQR)	6.7 (4.7–8.4)
C-reactive protein, mg/L; (median, IQR)	79 (51–102)
White blood cell, 10^9^/L;	1.8 (1.5–2.2)
Hepatic dysfunction, No. (%)	57 (54.8)
Kidney dysfunction, No. (%)	25 (24)
Respiratory dysfunction, No. (%)	29 (27.9)
SOFA scores (median, IQR)	4 (3–5)
Septic shock, No. (%)	20 (19.2)
**Condition after infections control**
Required number of drainage tubes (median, IQR)	2 (2–3)
Hemoglobin, g/L; (median, IQR)	90 (81–99)
Albumin, g/L; (median, IQR)	34 (32–36)
Platelet, 10^9^/L; (median, IQR)	157 (126–194)
C-reactive protein, mg/L; (median, IQR)	23 (15–30)
White blood cell, 10^9^/L;	0.7 (0.5–0.8)
Required intermittent blood transfusions, No. (%)	5 (4.8)
Required intermittent albumin transfusions, No. (%)	13 (12.5)
Required intermittent renal replacement therapy, No. (%)	4 (3.8)
**Comorbidity, No. (%)**
Hypertension	6 (5.8)
Diabetes	3 (2.9)

### 3.2 The 90-days closure rate

**Figure 1:**
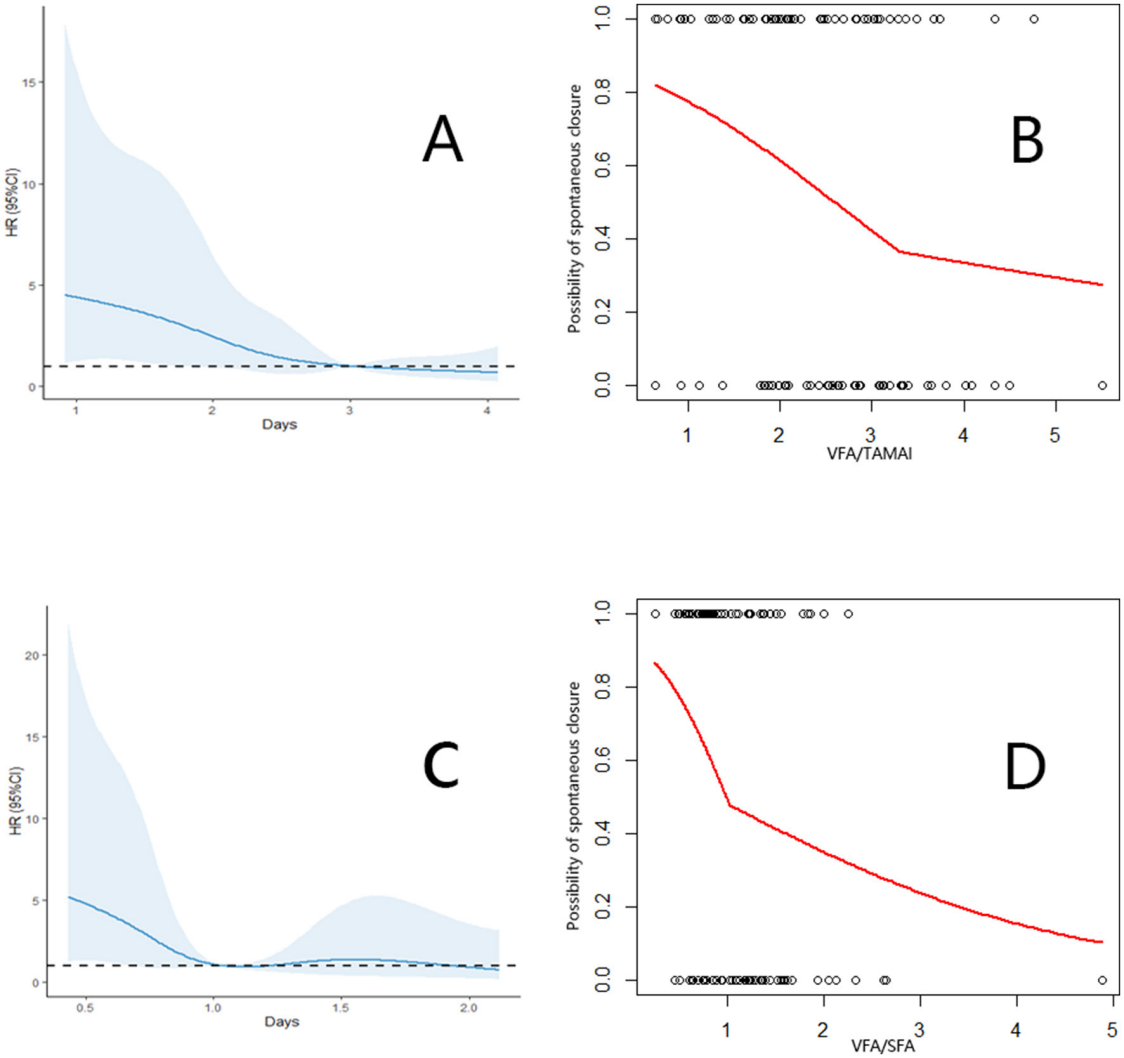
**(A)** Association between the VFA/TAMAI and spontaneous closure. **(B)** Cut off of the VFA/TAMAI using the segmented analysis. **(C)** Association between the VFA/SFA and spontaneous closure. **(D)** Cut off of the VFA/SFA using the segmented analysis.

**Figure 2:**
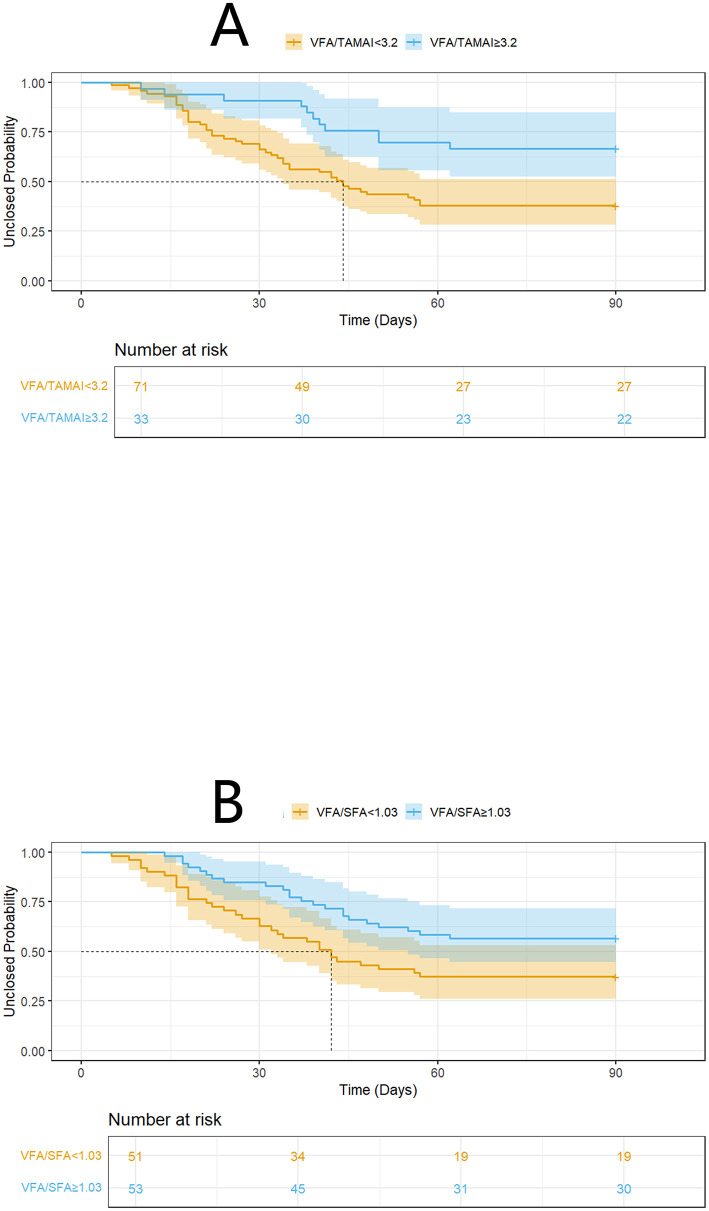
K-M analysis on spontaneous closure according to VFA/TAMAI **(A)**, and VFA/SFA **(B)**.

Spontaneous closure within 90 days occurred in 55 patients (52.9%), with a median time to closure of 31 days (range: 18–41 days) after infection control. The VFA, TAMAI, and SFA values for the entire cohort are presented in Table 2. Supplementary Table 1 revealed the characteristics between patients with or without closure. Using the Cox proportional hazards model, the optimal cutoff for VFA/TAMAI was determined to be 3.20 (Figures 1A, B). The population of patients with VFA/TAMAI ≥3.20 comprised 33 individuals (31.7%). Kaplan-Meier analysis revealed that patients with a lower VFA/TAMAI (< 3.20) had a higher incidence of spontaneous closure (Log-rank *P* = 0.005, Figure 2A). The optimal cutoff for VFA/SFA was 1.03 (Figures 1C, D). There were 53 patients (50.9%) with VFA/SFA ≥1.03. Kaplan-Meier analysis indicated that VFA/SFA < 1.03 was associated with a higher incidence of spontaneous closure (Log-rank *P* = 0.02, Figure 2B). The adjusted Cox regression model is presented in Table 3. The results suggest that VFA/TAMAI (HR = 0.59; 95% CI: 0.42–0.85; *P* = 0.004) was associated with spontaneous closure, whereas VFA/SFA was not a predictive factor for closure.

**Table 2:** Characteristics of the body composition.

**Characteristics**	**Overall population**
Visceral fat area, cm^2^ (median, IQR)	75.8 (55.3–89.0)
Subcutaneous fat area, cm^2^ (median, IQR)	70.1 (53.3–93.5)
Total abdominal muscle area index, cm^2^/m^2^ (median, IQR)	27.2 (25.5–31.6)
VFA/TAMAI	2.59 (1.96–3.31)
VFA/SFA	1.03 (0.75–1.43)

**Table 3:** Cox regression analysis for spontaneous closure.

**Characteristics**	**Unadjusted**			**Adjusted**		
	**HR**	**95%CI**	***P***	**HR**	**95%CI**	***P***
Male	0.75	0.44–1.28	0.29			
Age	0.99	0.98–1.01	0.81			
BMI	1.01	0.88–1.14	0.98			
**Location**						
Stump	Ref					
Bulb	1.09	0.58–2.06	0.77			
Descending part	0.84	0.41–1.71	0.63			
Horizontal part	0.58	0.15–1.25	0.21			
**Etiology**						
Tumor	Ref					
Ulcer	1.06	0.54–2.09	0.85			
Trauma	0.87	0.46–1.67	0.68			
Others	0.47	0.11–2.04	0.32			
**Output**						
< 500 mL/day	Ref					
≥500 mL/day, and < 1,000 mL/day	0.58	0.22–1.55	0.28			
≥1,000 mL/day	0.46	0.18–1.19	0.11			
Interval from fistula occurred to infection control	0.96	0.93–0.99	0.040	0.95	0.92–0.99	0.010
Infection invading the retroperitoneum	0.68	0.40–1.16	0.16			
Requirement for emergency laparotomy	0.61	0.31–1.19	0.14			
Abdominal bleeding occurred during the treatment process	0.64	0.35–1.18	0.15			
Endoscopic fistula diameter >2 cm	0.39	0.16–0.92	0.032	0.38	0.16–0.93	0.034
Hemoglobin on admission	1.01	0.97–1.11	0.56			
Albumin on admission	1.01	0.92–1.06	0.67			
Platelet on admission	1.00	0.99–1.01	0.31			
Procalcitonin on admission	0.98	0.74–1.13	0.95			
C-reactive protein on admission	0.99	0.99–1.01	0.87			
White blood cell on admission	0.98	0.53–1.80	0.94			
Hepatic dysfunction on admission	0.72	0.42–1.23	0.23			
Kidney dysfunction on admission	0.74	0.38–1.43	0.37			
Respiratory dysfunction on admission	0.91	0.49–1.67	0.76			
SOFA scores on admission	0.98	0.86–1.10	0.71			
Septic shock on admission	1.17	0.60–2.26	0.65			
Required number of drainage tubes after infections control	0.73	0.52–1.29	0.18			
Hemoglobin after infections control	1.01	0.97–1.11	0.65			
Albumin after infections control	1.00	0.90–1.11	0.98			
Platelet after infections control	1.01	0.99–1.01	0.14			
C-reactive protein after infections control	0.97	0.95–1.00	0.050	0.99	0.96–1.02	0.48
White blood cell after infections control	0.61	0.16–2.35	0.48			
VFA/TAMAI ≥3.2 after infections control	0.40	0.21–0.78	0.007	0.59	0.42–0.85	0.004
VFA/SFA ≥ 1.03 after infections control	0.45	0.26–0.81	0.008	0.61	0.33–1.17	0.12
Required intermittent blood transfusions after infections control	0.62	0.15–2.55	0.51			
Required intermittent albumin transfusionsafter infections control	2.86	1.05–4.16	0.037	2.50	1.23–5.08	0.011
Required intermittent renal replacement therapyafter infections control	0.34	0.05–2.42	0.28			
Hypertension	0.99	0.31–3.19	0.96			
Diabetes	0.51	0.07–3.69	0.51			

### 3.3 Cox- model analysis

**Figure 3:**
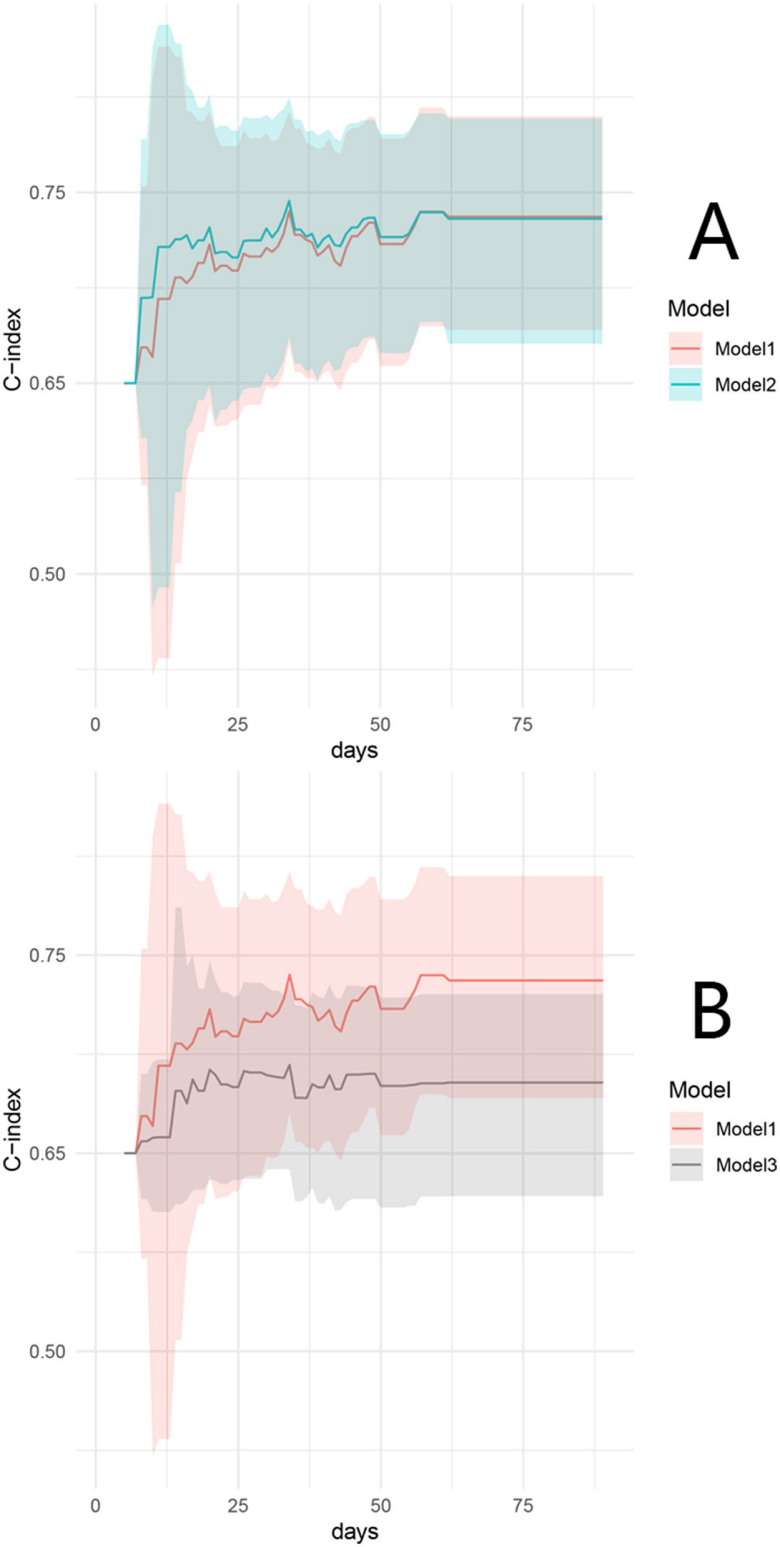
Time–C-index curve between model 1 and model 2 **(A)**, and model 1 and model 3 **(B)**.

The Cox model, including the factors of the interval from fistula occurrence to infection control, endoscopic fistula diameter >2 cm, VFA/TAMAI ≥3.20 after infection control, and the requirement for intermittent albumin transfusions after infection control, was defined as Model 1. A second model, with VFA/SFA ≥1.03 added, was defined as Model 2. Discrimination analysis revealed that the 90-day C-index for Model 1 was 0.73 (95% CI: 0.67–0.78), with a bootstrap-corrected C-index of 0.72. Model 2 had a C-index of 0.73 (95% CI: 0.68–0.78), with a bootstrap-corrected C-index of 0.72. Figure 3A shows the comparable bootstrap-corrected time-C-index curves between the two models (*P* = 0.74). In contrast, we excluded VFA/TAMAI ≥3.20 from Model 1, defining this as Model 3. The 90-day C-index for Model 3 was 0.67 (95% CI: 0.63–0.72), with a bootstrap-corrected C-index of 0.67. Figure 3B illustrates the bootstrap-corrected time-C-index curve between the two models (*P* = 0.013). These results suggest that VFA/TAMAI, rather than VFA/SFA, influences spontaneous closure.

### 3.4 Sensitivity analyses

**Figure 4:**
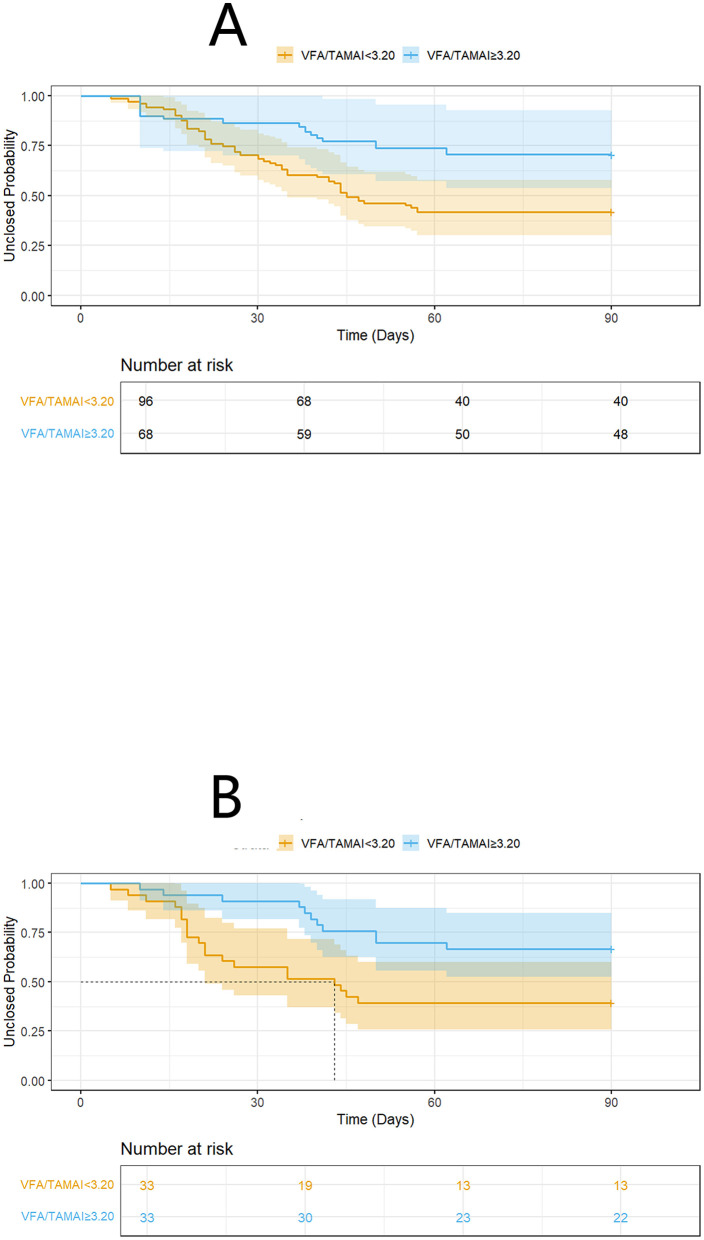
K-M analysis on spontaneous closure according to VFA/TAMAI after IPTW **(A)**, and PSM **(B)**.

We conducted additional sensitivity analyses. The characteristics of patients with differing VFA/TAMAI ratios are presented in Table 4. An inverse probability of treatment weighting (IPTW) approach generated 151.3 pseudo-individuals (Table 5) to address baseline imbalances between groups defined by VFA/TAMAI. Notably, the prognostic significance of VFA/TAMAI ≥3.20 remained evident (HR = 0.38; 95% CI: 0.17–0.84; *P* = 0.016) after adjusting for variables with a standardized mean difference (SMD)>0.2 (Figure 4A; log-rank *P* = 0.014). In addition, 66 matched cases were identified via propensity score matching (PSM), with characteristics shown in Table 6. VFA/TAMAI ≥3.20 was the only factor significantly associated with spontaneous closure (HR = 0.39; 95% CI: 0.19–0.83; *P* = 0.014; Figure 4B; log-rank *P* = 0.011).

**Table 4:** Characteristics between patients with different VFA/TAMAI.

**Characteristics**	**VFA/TAMAI ≥3.2**	**VFA/TAMAI < 3.2**	***P***
VFA/SFA≥1.03, No. (%)	24 (72.3)	29 (40.8)	0.002
**Demographic data**			
Male, No. (%)	18 (54.5)	46 (64.8)	0.32
Age, years (median, IQR)	50 (37–62)	46 (35–63)	0.61
BMI, kg/m^2^ (median, IQR)	19.8 (19.2–21.5)	20.3 (18.1–22.1)	0.85
**Fistula characteristics**			
**Location, No. (%)**			0.88
Stump	14 (42.4)	27 (38.1)	
Bulb	11 (33.3)	22 (30.9)	
Descending part	7 (21.2)	18 (25.4)	
Horizontal part	1 (3.1)	4 (5.6)	
**Etiology, No. (%)**			0.99
Tumor	11 (33.3)	22 (30.9)	
Ulcer	9 (27.3)	19 (26.8)	
Trauma	11 (33.3)	25 (35.2)	
Others	2 (6.1)	5 (7.1)	
**Output**			0.80
< 500 mL/day	3 (9.1)	4 (5.6)	
≥500 mL/day, and < 1,000 mL/day	11 (33.3)	24 (33.8)	
≥1,000 mL/day	19 (57.6)	43 (60.6)	
Interval from fistula occurred to admission, days (median, IQR)	20 (12–22)	18 (12–22)	0.44
Interval from fistula occurred to infection control, days (median, IQR)	40 (33–45)	38 (31–44)	0.62
Infection invading the retroperitoneum, No. (%)	15 (45.5)	40 (56.3)	0.30
Requirement for emergency laparotomy, No. (%)	14 (42.4)	18 (25.4)	0.015
Abdominal bleeding occurred during the treatment process, No. (%)	16 (48.5)	18 (25.4)	0.019
Endoscopic fistula diameter >2 cm, No. (%)	6 (18.2)	11 (15.5)	0.73
**Condition on admission**			
Hemoglobin, g/L (median, IQR)	77 (63–91)	83 (64–91)	0.35
Albumin, g/L (median, IQR)	29 (25–33)	29 (26–32)	0.98
Platelet, 10^9^/L (median, IQR)	90 (69–149)	108 (81–152)	0.26
Procalcitonin, ng/mL (median, IQR)	7.1 (5.3–8.8)	6.4 (4.5–8.3)	0.52
C-reactive protein, mg/L (median, IQR)	85 (51–106)	78 (51–100)	0.56
White blood cell, 10^9^/L;	1.8 (1.6–2.2)	1.7 (1.4–2.2)	0.55
Hepatic dysfunction, No. (%)	23 (69.7)	34 (47.9)	0.038
Kidney dysfunction, No. (%)	7 (21.1)	18 (25.4)	0.65
Respiratory dysfunction, No. (%)	13 (39.4)	16 (22.5)	0.074
SOFA scores (median, IQR)	5 (4–7)	4 (2–5)	0.015
Septic shock, No. (%)	9 (27.3)	11 (15.5)	0.16
**Condition after infections control**			
Required number of drainage tubes (median, IQR)	2 (2−3)	2 (2−3)	0.28
Hemoglobin, g/L (median, IQR)	86 (80–98)	91 (81–100)	0.42
Albumin, g/L (median, IQR)	34 (32–36)	34 (31–36)	0.86
Platelet, 10^9^/L (median, IQR)	151 (115–196)	159 (132–192)	0.41
C-reactive protein, mg/L (median, IQR)	27 (18–33)	23 (14–29)	0.022
White blood cell, 10^9^/L;	0.7 (0.5–0.9)	0.7 (0.5–0.8)	0.33
Required intermittent blood transfusions, No. (%)	3 (9.1)	2 (2.8)	0.16
Required intermittent albumin transfusions, No. (%)	8 (24.2)	5 (7.0)	0.014
Required intermittent renal replacement therapy, No. (%)	2 (6.1)	2 (2.8)	0.43
**Comorbidity, No. (%)**			
Hypertension	2 (6.1)	4 (5.6)	0.93
Diabetes	2 (6.1)	1 (1.4)	0.18

**Table 5:** Characteristics between pseudo-individuals in different VFA/TAMAI.

**Characteristics**	**VFA/TAMAI ≥3.2**	**VFA/TAMAI < 3.2**	**SMD**
The number of pseudo-individuals after IPTW matching	60.6	90.7	N/A
VFA/SFA≥1, No. (%)	39.8 (65.7)	41.7 (45.9)	0.41
**Demographic data**			
Male, No. (%)	39.2 (64.7)	59 (65.8)	0.023
Age, years [mean (SD)]	49.86 (18.16)	38.24 (7.99)	0.005
BMI, kg/m^2^ [mean (SD)]	20.25 (1.47)	20.20 (2.06)	0.027
**Fistula characteristics**			
**Location, No. (%)**			0.040
Stump	22.8 (37.6)	33.7 (37.1)	
Bulb	18.0 (29.7)	27.5 (30.3)	
Descending part	16.6 (27.4)	24.0 (26.4)	
Horizontal part	3.3 (5.4)	5.6 (6.2)	
**Etiology, No. (%)**			0.092
Tumor	18.8 (31.0)	27.4 (30.2)	
Ulcer	18.1 (29.9)	25.4 (28.0)	
Trauma	18.3 (30.2)	31.0 (34.2)	
Others	5.4 (8.9)	6.9 (7.6)	
**Output**			0.101
< 500 mL/day	4.4 (7.2)	5.2 (5.8)	
≥500 mL/day, and < 1,000 mL/day	23.6 (38.9)	32.3 (35.6)	
≥1,000 mL/day	32.7 (53.9)	53.2 (58.6)	
Interval from fistula occurred to admission, days [mean (SD)]	19 (4.61)	19 (4.56)	0.004
Interval from fistula occurred to infection control, days [mean (SD)]	38.28 (7.58)	38.24 (7.99)	0.005
Infection invading the retroperitoneum, No. (%)	30.1 (49.6)	51.5 (56.8)	0.15
Requirement for emergency laparotomy, No. (%)	21.4 (23.6)	21.9 (36.2)	0.28
Abdominal bleeding occurred during the treatment process, No. (%)	28.0 (46.2)	26.2 (28.9)	0.36
Endoscopic fistula diameter >2 cm, No. (%)	12.0 (19.8)	15.1 (16.6)	0.084
**Condition on admission**			
Hemoglobin, g/L [mean (SD)]	79.86 (16.73)	84.00 (15.26)	0.26
Albumin, g/L [mean (SD)]	28.98 (4.01)	29.12 (4.14)	0.035
Platelet, 10^9^/L [mean (SD)]	107.89 (52.12)	112.05 (45.42)	0.085
Procalcitonin, ng/mL [mean (SD)]	6.84 (1.75)	6.64 (1.77)	0.091
C-reactive protein, mg/L [mean (SD)]	81.73 (27.42)	78.27 (27.16)	0.13
White blood cell, 10^9^/L;	1.88 (0.46)	1.85 (0.41)	0.073
Hepatic dysfunction, No. (%)	46.7 (51.4)	40.2 (66.2)	0.30
Kidney dysfunction, No. (%)	16.1 (26.6)	23.1 (25.4)	0.027
Respiratory dysfunction, No. (%)	14.8 (24.4)	23 (25.4)	0.023
SOFA scores (median, IQR)	5 (2.14)	4 (2.13)	0.23
Septic shock, No. (%)	10.1 (16.7)	14.5 (16.0)	0.020
**Condition after infections control**			
Required number of drainage tubes [mean (SD)]	2.23 (0.74)	2.30 (0.80)	0.092
Hemoglobin, g/L [mean (SD)]	87.91 (12.43)	89.39 (10.86)	0.13
Albumin, g/L [mean (SD)]	34.11 (2.57)	33.97 (2.36)	0.056
Platelet, 10^9^/L [mean (SD)]	154.55 (47.49)	159.30 (40.75)	0.11
C-reactive protein, mg/L [mean (SD)]	25.37 (9.71)	22.25 (9.12)	0.33
White blood cell, 10^9^/L;	0.72 (0.20)	0.67 (0.20)	0.27
Required intermittent blood transfusions, No. (%)	4.4 (7.2)	3.4 (3.8)	0.15
Required intermittent albumin transfusions, No. (%)	8.3 (13.7)	9.2 (10.1)	0.11
Required intermittent renal replacement therapy, No. (%)	2 (3.3)	2 (2.2)	0.43
**Comorbidity, No. (%)**			
Hypertension	3.1 (5.1)	5.2 (5.7)	0.029
Diabetes	2.3 (3.8)	1.4 (1.6)	0.15

**Table 6:** Characteristics between patients in different VFA/TAMAI after PSM.

**Characteristics**	**VFA/TAMAI ≥3.2**	**VFA/TAMAI < 3.2**	***P***
VFA/SFA≥1.03, No. (%)	24 (72.3)	17 (51.5)	0.079
**Demographic data**			
Male, No. (%)	18 (54.5)	21 (63.6)	0.45
Age, years [mean (SD)]	50 (37–62)	50 (35–63)	0.91
BMI, kg/m^2^ [mean (SD)]	19.8 (19.2–21.5)	20.6 (18.4–22.0)	0.71
**Fistula characteristics**			
**Location, No. (%)**			0.76
Stump	14 (42.4)	17 (51.5)	
Bulb	11 (33.3)	8 (24.2)	
Descending part	7 (21.2)	6 (18.2)	
Horizontal part	1 (3.1)	2 (6.1)	
**Etiology, No. (%)**			0.78
Tumor	11 (33.3)	14 (42.4)	
Ulcer	9 (27.3)	8 (24.2)	
Trauma	11 (33.3)	8 (24.2)	
Others	2 (6.1)	3 (9.1)	
**Output**			0.72
< 500 mL/day	3 (9.1)	2 (6.1)	
≥500 mL/day, and < 1,000 mL/day	11 (33.3)	14 (42.4)	
≥1,000 mL/day	19 (57.6)	17 (51.5)	
Interval from fistula occurred to admission, days [mean (SD)]	40 (33–45)	20 (12–22)	0.72
Interval from fistula occurred to infection control, days [mean (SD)]	20 (12–22)	41 (32–45)	0.61
Infection invading the retroperitoneum, No. (%)	15 (45.5)	13 (39.4)	0.62
Requirement for emergency laparotomy, No. (%)	14 (42.4)	11 (33.3)	0.45
Abdominal bleeding occurred during the treatment process, No. (%)	16 (48.5)	12 (36.4)	0.32
Endoscopic fistula diameter >2 cm, No. (%)	6 (18.2)	7 (21.1)	0.76
**Condition on admission**			
Hemoglobin, g/L [mean (SD)]	77 (63–91)	83 (68–95)	0.25
Albumin, g/L [mean (SD)]	29 (25–33)	29 (26–32)	0.89
Platelet, 10^9^/L [mean (SD)]	90 (69–81)	98 (62–138)	0.53
Procalcitonin, ng/mL [mean (SD)]	7.1 (5.3–8.8)	6.6 (4.7–8.4)	0.81
C-reactive protein, mg/L [mean (SD)]	85 (51–106)	85 (61–102)	0.99
White blood cell, 10^9^/L;	1.8 (1.6–2.2)	1.8 (1.6–2.2)	0.93
Hepatic dysfunction, No. (%)	23 (69.7)	19 (57.6)	0.31
Kidney dysfunction, No. (%)	7 (21.1)	8 (24.2)	0.77
Respiratory dysfunction, No. (%)	13 (39.4)	8 (24.2)	0.19
SOFA scores (median, IQR)	5 (4–7)	4 (3-5)	0.40
Septic shock, No. (%)	9 (27.3)	4 (12.1)	0.12
**Condition after infections control**			
Required number of drainage tubes [mean (SD)]	2 (2−3)	2 (2–3)	0.58
Hemoglobin, g/L [mean (SD)]	86 (80–98)	90 (82–99)	0.46
Albumin, g/L [mean (SD)]	34 (32–36)	35 (33–36)	0.52
Platelet, 10^9^/L [mean (SD)]	151 (115–196)	156 (126–192)	0.75
C-reactive protein, mg/L [mean (SD)]	27 (18–33)	22 (15–29)	0.18
White blood cell, 10^9^/L;	0.7 (0.5–0.9)	0.7 (0.5–0.8)	0.57
Required intermittent blood transfusions, No. (%)	3 (9.1)	1 (3.0)	0.31
Required intermittent albumin transfusions, No. (%)	8 (24.2)	3 (9.1)	0.10
Required intermittent renal replacement therapy, No. (%)	2 (6.1)	1 (3.0)	0.56
**Comorbidity, No. (%)**			
Hypertension	2 (6.1)	1 (3.0)	0.56
Diabetes	2 (6.1)	1 (3.0)	0.56

## 4 Discussion

This study provides novel insights into the prognostic value of body composition parameters for spontaneous closure in patients with duodenal fistulas following sepsis resolution. Our findings demonstrate that VFA/TAMAI is a significant predictor of spontaneous closure, with lower ratios (< 3.20) associated with higher closure likelihood. Although the VFA/SFA ratio exhibited predictive value in initial analyses, it did not retain statistical significance in multivariate models.

These findings suggest that the balance between visceral fat and muscle mass may be more critical for healing outcomes than fat distribution alone. The diminished predictive value of VFA/SFA in multivariate analysis—despite its initial significance—underscores the complex interplay between adipose depots and muscle mass in health outcomes.

While visceral adiposity is a recognized risk factor for metabolic disease and inflammation, emerging evidence highlights the pivotal role of muscle mass when considered alongside fat metrics. Visceral adipose tissue (VAT) is metabolically active, secreting pro-inflammatory cytokines such as interleukin-6 (IL-6) and tumor necrosis factor-alpha (TNF-α), thereby contributing to systemic inflammation and insulin resistance. In contrast, subcutaneous adipose tissue (SAT), especially in the gluteofemoral region, has protective metabolic effects, acting as a lipid buffer to prevent lipotoxicity in non-adipose tissues.

Incorporating muscle mass into the assessment via the VFA-to-muscle ratio adds an important dimension to evaluating metabolic health. Skeletal muscle is crucial not only for mobility but also for glucose uptake and metabolism. It secretes myokines, such as irisin and myostatin, which exert systemic effects on metabolism and inflammation. Sarcopenia—defined as the loss of muscle mass and function—is associated with increased morbidity and mortality across various populations.

For example, Lieffers et al. (18) reported that in patients undergoing colorectal cancer surgery, sarcopenia independently predicted higher rates of postoperative infections and prolonged hospital stays, whereas adiposity was not a significant predictor. In chronic inflammatory diseases such as Crohn's disease, muscle loss may exacerbate disease severity. Zhang et al. (19) found that Crohn's patients with sarcopenia had elevated inflammatory markers and poorer outcomes compared to those without sarcopenia. Muscle depletion may impair immunity and wound healing, adversely affecting prognosis.

Nutritional interventions can further complicate associations between fat distribution and outcomes. In patients receiving nutritional support, lean mass is often restored preferentially over fat. Weijs et al. (20) found that adequate protein intake preserved muscle mass in critically ill patients, with limited effects on fat mass. This preferential recovery may modulate body composition in ways that reduce the apparent impact of fat-based indices.

In oncology, similar trends have been observed. Prado et al. (21) reported that patients with metastatic colorectal cancer and sarcopenic obesity (high fat, low muscle mass) had poorer survival compared to those with normal muscle mass, regardless of fat content. Moreover, chemotherapy-related toxicity was more severe in sarcopenic patients, underscoring the importance of muscle in treatment tolerance.

Biological mechanisms may underpin the strong influence of muscle mass. Skeletal muscle contributes to systemic inflammation modulation through anti-inflammatory cytokines and myokines (11). Adequate muscle also promotes metabolic homeostasis by enhancing insulin sensitivity and lipid oxidation, potentially counteracting the detrimental effects of visceral adiposity.

This interrelationship is also highlighted in the adipose tissue expandability hypothesis, which posits that individuals with higher SAT expansion capacity can buffer ectopic fat deposition and its metabolic complications (22). Conversely, limited SAT expandability leads to VAT accumulation and dysfunction. Thus, SAT and its ratio to VAT and muscle mass is crucial in determining metabolic health (23).

In conclusion, the diminished prognostic value of VFA/SFA in multivariate analyses may reflect the overriding influence of muscle mass and the protective role of SAT. Muscle loss appears to be a dominant mediator of poor outcomes, outweighing fat distribution effects when both are considered. Future studies should incorporate comprehensive body composition assessments—including muscle mass and regional fat distribution—to better predict clinical outcomes and tailor therapeutic strategies.

Several limitations must be acknowledged. First, the retrospective design introduces selection bias, particularly concerning variations in nutrition and drainage protocols across centers. Second, body composition was assessed only at sepsis resolution, potentially overlooking dynamic changes. Third, the modest sample size (*n* = 104) limits subgroup analyses, such as comparisons between trauma- and tumor-related fistulas. Additionally, our study lacks malnutrition assessment tools. In Petra G's latest study (24), most assessment tools have predictive value for the prognosis of abdominal surgery, with the Malnutrition Universal Screening Tool (MUST) identified as the most valuable. For surgical patients, these tools could be incorporated in future studies to optimize research strategies. Future work should also include functional measures (e.g., handgrip strength) and mechanistic studies examining adipomyokines (e.g., myostatin, leptin) to elucidate the pathways linking VFA/TAMAI to mucosal healing.

## 5 Conclusion

Our study highlights the potential of the VFA/TAMAI ratio as a prognostic indicator for spontaneous closure in duodenal fistula patients recovering from sepsis. These findings not only contribute to our understanding of the complex interplay between body composition and healing processes but also suggest potential avenues for personalized management strategies in this challenging patient population.

## Data Availability

The raw data supporting the conclusions of this article will be made available by the authors, without undue reservation.
