# Supplementary material for: Visceral fat-to-muscle ratio guides spontaneous closure in post-sepsis duodenal fistula: a body composition divergence beyond visceral-subcutaneous fat ratio
Source: Front Nutr. 2025 May 30;12:1604230. doi: 10.3389/fnut.2025.1604230 (PMC12162335; doi:10.3389/fnut.2025.1604230)
Supplement: Supplementary file 1 [file Table_1.doc]

Supplementary Table 1. Characteristics categorized by the occurrence of spontaneous closure

| Characteristics | Spontaneous closure  (n = 55) | Non - closure  (n = 49) | *P* |
| --- | --- | --- | --- |
| VFA/TAMAI ≥3.2, No. (%) | 11 (20) | 22 (44.9) | 0.006 |
| VFA/SFA ≥1.03, No. (%) | 23 (41.8) | 30 (61.2) | 0.048 |
| Demographic data |  |  |  |
| Male,No. (%) | 31 (56.4) | 33 (67.3) | 0.25 |
| Age, years;(median,IQR ) | 46 (35 - 63) | 50 (37 - 61) | 0.79 |
| BMI, kg/m2,(median,IQR ) | 20.4 (18.1 - 21.9) | 19.8 (18.8 - 22.2) | 0.84 |
| Fistula characteristics |  |  |  |
| Location, No. (%) |  |  | 0.62 |
| Stump | 21 (38.2) | 20 (40.8) |  |
| Bulb | 18 (32.7) | 15 (30.6) |  |
| Descending part | 12 (21.8) | 13 (26.5) |  |
| Horizontal part | 4 (7.3) | 1 (2.1) |  |
| Etiology,No. (%) |  |  |  |
| Tumor | 18 (32.7) | 15 (30.6) | 0.59 |
| Ulcer | 16 (29.1) | 12 (2.5) |  |
| Trauma | 19 (34.6) | 17 (34.7) |  |
| Others | 2 (3.6) | 5 (10.2) |  |
| Output |  |  | 0.42 |
| <500mL/day | 5 (9.1) | 2 (4.1) |  |
| ≥ 500mL/day, and <1000mL/day | 20 (36.4) | 15 (30.6) |  |
| ≥ 1000mL/day | 30 (54.5) | 32 (6.5) |  |
| Interval from fistula occurred to admission, days, (median,IQR ) | 19 (11 - 22) | 19 (13 - 22) | 0.39 |
| Interval from fistula occurred to infection control, days, (median,IQR ) | 38 (31 - 44) | 40 (32 - 45) | 0.12 |
| Infection invading the retroperitoneum, No. (%) | 26 (47.3) | 29 (59.2) | 0.22 |
| Requirement for emergency laparotomy, No. (%) | 14 (25.5) | 18 (36.7) | 0.21 |
| Abdominal bleeding occurred during the treatment process, No. (%) | 14 (25.5) | 20 (40.8) | 0.096 |
| Endoscopic fistula diameter greater than 2cm, No. (%) | 6 (10.9) | 11 (22.4) | 0.11 |
| Condition on admission |  |  |  |
| Hemoglobin, g/L; (median,IQR ) | 81 (63 - 92) | 78 (63 - 91) | 0.53 |
| Albumin, g/L; (median,IQR ) | 29 (26 - 33) | 29 (25 - 32) | 0.44 |
| Platelet, 10^9^/L; (median,IQR ) | 102 (81 - 157) | 98 (73 - 147) | 0.31 |
| Procalcitonin, ng/mL; (median,IQR ) | 6.6 (4.5 - 8.2) | 6.9 (4.6 - 8.6) | 0.71 |
| C-reactive protein, mg/L; (median,IQR ) | 76 (52 - 105) | 82 (50 - 102) | 0.86 |
| White blood cell, 10^9^/L; | 1.7 (1.4 - 2.1) | 1.8 (1.5 - 2.2) | 0.91 |
| Hepatic dysfunction,No. (%) | 28 (50.9) | 29 (59.2) | 0.39 |
| Kidney dysfunction,No. (%) | 11 (20) | 14 (28.6) | 0.31 |
| Respiratory dysfunction,No. (%) | 14 (25.5) | 15 (30.6) | 0.56 |
| SOFA scores (median,IQR ) | 4 (2 - 5) | 5 (3 - 6) | 0.027 |
| Septic shock,No. (%) | 10 (18.2) | 10 (20.4) | 0.83 |
| Condition after infections control |  |  |  |
| Required number of drainage tubes (median,IQR ) | 2 (2 -3) | 2 (2 -3) | 0.31 |
| Hemoglobin, g/L; (median,IQR ) | 90 (81 - 99) | 90 (80 - 100) | 0.68 |
| Albumin, g/L; (median,IQR ) | 34 (32 - 36) | 34 (31 - 36) | 0.99 |
| Platelet, 10^9^/L; (median,IQR ) | 169 (136 - 194) | 148 (119 - 195) | 0.15 |
| C-reactive protein, mg/L; (median,IQR ) | 21 (13 - 30) | 24 (18 - 31) | 0.11 |
| White blood cell, 10^9^/L; | 0.7 (0.5 - 0.9) | 0.7 (0.5 - 0.9) | 0.59 |
| Required intermittent blood transfusions, No. (%) | 2 (3.6) | 3 (6.1) | 0.55 |
| Required intermittent albumin transfusions, No. (%) | 5 (9.1) | 8 (16.3) | 0.26 |
| Required intermittent renal replacement therapy, No. (%) | 1 (18.2) | 3 (6.1) | 0.49 |
| Comorbidity, No. (%) |  |  |  |
| Hypertension | 3 (5.5) | 3 (6.1) | 0.88 |
| Diabetes | 1 (18.2) | 2 (4.1) | 0.49 |
